# Carbon Nanotubes as Supports for Inulinase Immobilization

**DOI:** 10.3390/molecules190914615

**Published:** 2014-09-15

**Authors:** Tais B. Garlet, Caroline T. Weber, Rodrigo Klaic, Edson L. Foletto, Sergio L. Jahn, Marcio A. Mazutti, Raquel C. Kuhn

**Affiliations:** Chemical Engineering, Federal University of Santa Maria, 97105-900 Santa Maria, Brazil

**Keywords:** inulinase, immobilization, multiwalled carbon nanotubes, experimental design

## Abstract

The commercial inulinase obtained from *Aspergillus niger* was non-covalently immobilized on multiwalled carbon nanotubes (MWNT-COOH). The immobilization conditions for the carbon nanotubes were defined by the central composite rotational design (CCRD). The effects of enzyme concentration (0.8%–1.7% v/v) and adsorbent:adsorbate ratio (1:460–1:175) on the enzyme immobilization were studied. The adsorbent:adsorbate ratio variable has positive effect and the enzyme concentration has a negative effect on the inulinase immobilization (U/g) response at the 90% significance level. These results show that the lower the enzyme concentration and the higher the adsorbent:adsorbate ratio, better is the immobilization. According to the results, it is possible to observe that the carbon nanotubes present an effective inulinase adsorption. Fast adsorption in about six minutes and a loading capacity of 51,047 U/g support using a 1.3% (v/v) inulinase concentration and a 1:460 adsorbent:adsorbate ratio was observed. The effects of temperature on the immobilized enzyme activity were evaluated, showing better activity at 50 °C. The immobilized enzyme maintained 100% of its activity during five weeks at room temperature. The immobilization strategy with MWNT-COOH was defined by the experimental design, showing that inulinase immobilization is a promising biotechnological application of carbon nanotubes.

## 1. Introduction

Enzymes have been used in various industrial processes, such as the production of pharmaceuticals, foods, biosensors and biofuels. As a result, nowadays, interest in enzyme immobilization has increased and it has been successfully applied in many processes [1]. Enzymes can be immobilized on solid and insoluble supports, which results in an improvement in some properties, among them, the thermal stabilities, recovery, and so, the technique can be applied in many industrial processes, making possible enzyme reuse and thus reducing costs [2,3]. Immobilization can also provide additional advantages such as lowering downstream purification requirements because the products can be easily removed from the immobilized enzymes [4].

Inulinases are potentially useful enzymes for the production of sugars (fructose and fructooligosaccharides). Inulinase produces high fructose syrups by enzymatic hydrolysis with yields of about 95% [5], and they are enzymes widely used for the production of fructooligosaccharides, which are functional food ingredients that may be employed in product formulations for use in low-calorie diets with nutritional properties, such as low cariogenicity, and higher sweetening capacity than sucrose [6].

Nanoparticles are excellent supports for enzyme immobilization because have high surface area and mechanical stability that are interesting and important characteristics for the immobilization process [7], but the recovery by centrifugation and filtration of these nanoparticles is very difficult, although the recovery could be improved and facilitated by using magnetic nanoparticles by exposure to a magnetic field and thus can be reused [8,9].

Nanostructured materials (nanoparticles, nanofibers, carbon nanotubes, *etc.*) have been receiving more attention from researchers in the last years [10]. Carbon nanotubes (CNTs) are nanometer scale graphitic sheets with diameters smaller than 100 nm [11]. Single-walled carbon nanotubes (SWNTs) and multiwalled carbon nanotubes (MWNTs) have been used for enzyme immobilization [10]. The non-porous nanoparticles have kinetic advantages compared to other immobilized biocatalysts, but some drawbacks can be observed, among them, the stabilization effects of the immobilization may be decreased or lost [9].

Non-covalent and covalent attachments have been reported for the immobilization of enzymes [3,12]. Covalent attachment is sometimes preferred because it maintains the enzyme activity, although occasionally the enzyme structure can be disrupted [3,10,12]. Non-covalent immobilization is mainly based on physical adsorption, whereby the enzyme is attached to the matrix through hydrogen bonding, van der Waals forces or hydrophobic interactions. The immobilization by adsorption usually preserves the catalytic activity of the enzyme [13], but the immobilized enzyme can be lost during use when the interactions are relatively weak [10,13] and the support can be reused. In the enzyme immobilization the cost of the support must be considered during the design of the biocatalyst and sometimes can result in an increase in the final cost of the biocatalyst [9].

Enzyme immobilization is a promising biotechnological application of CNTs. However, to the best of our knowledge there are no literature reports on the application of carbon nanotubes for the immobilization of inulinase, so this work demonstrates its importance for further application of inulinase in industrial processes. This study was based on immobilization of commercial inulinase from *Aspergillus niger* on multiwalled carbon nanotubes (MWNT) because in large-scale processes, immobilized enzymes are used to lower the process cost. In order to obtain a better immobilization, several immobilization parameters such as enzyme concentration and the adsorbent:adsorbate ratio were evaluated according to a central composite rotational design (CCRD). Thermal and storage stabilities of the resulting immobilized enzyme were also evaluated in this work.

## 2. Results and Discussion

### 2.1. Experimental Design

**Figure 1:**
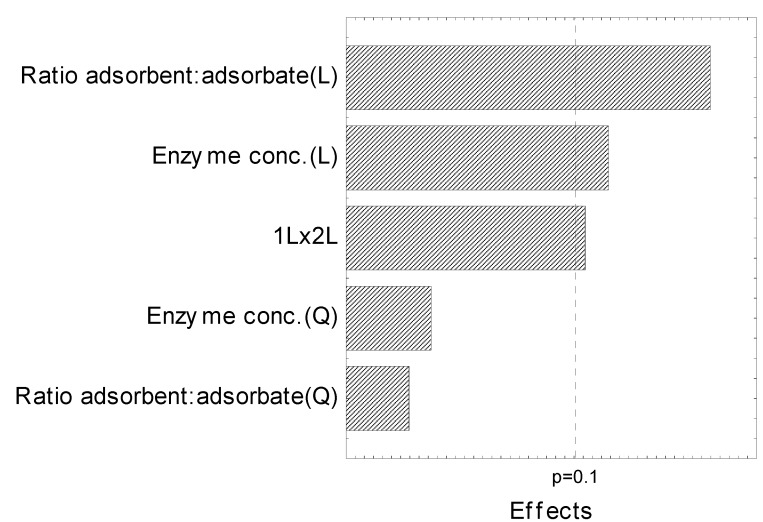
Pareto chart for the independent variables in the CCRD.

The conditions for better inulinase immobilization were studied through an experimental design (Table 1). The enzyme concentration and the adsorbent:adsorbate ratio that could influence the inulinase immobilization were studied and the effects of these variables were analyzed through CCRD. A Pareto chart (Figure 1) shows that the adsorbent:adsorbate ratio and enzyme concentration had a significant effect on the inulinase immobilization at the 90% confidence level (*p* < 0.1). The enzyme concentration and the adsorbent:adsorbate ratio have a negative effect on the inulinase immobilization response, showing that for best enzyme immobilization a lower inulinase concentration can be used. From the obtained results it is evident that as the adsorbent:adsorbate ratio decreases the immobilization yield increases.

**Table 1:** Experimental design, coded and real levels (in parentheses) and results of the inulinase immobilization.

Assays	Enzyme Concentration (%)	Ratio Adsorbent:Adsorbate	Immobilization (U/g)
1	−1 (1.0)	−1 (1:400)	45,774.44
2	1 (1.6)	−1 (1:400)	45,286.32
3	−1 (1.0)	1 (1:200)	49,570.90
4	1 (1.6)	1 (1:200)	26,665.60
5	−1.41 (0.87)	0 (1:300)	39,331.28
6	1.41 (1.7)	0 (1:300)	30,784.64
7	0 (1.3)	−1.41 (1:460)	51,047.76
8	0 (1.3)	1.41 (1:175)	24,603.97
9	0 (1.3)	0 (1:300)	38,715.53
10	0 (1.3)	0 (1:300)	40,217.43
11	0 (1.3)	0 (1:300)	40,175.71

According to the results (Table 1), it is possible to observe that the carbon nanotubes presented an effective inulinase adsorption, and a fast adsorption was observed after about six minutes in all assays and a loading capacity of 51,047 U/g support was observed using an inulinase concentration of 1.3% (v/v) and 1:460 of adsorbent:adsorbate ratio. In the same table is possible to verify the influence of the variables in the inulinase immobilization. Considering the enzyme concentration, at the intermediate concentrations a decrease in the adsorbent:adsorbate ratio improved the inulinase immobilization (assays 7, 8 and 9). An increase in the inulinase immobilization was observed when the adsorbent:adsorbate ratio was maintained at a constant rate and the enzyme concentration was decreased (assays 5 and 6).

L: linear and Q: quadratic parameters.

Inulinase activity retention by MWNTs may be due to the efficient heat conductivity of the CNTs. The MWNT conductivity can promote the heat transfer between substances [14]. Ji* et al.* [14] found that a high percentage of the enzyme activity was retained by MWNT, and the hydrolytic activity of MWNT-lipase found by these researchers was higher than that by the enzymes immobilized on single-walled CNTs in the work of Asuri* et al.* [15].

Results obtained in the CCRD were used to build quadratic models expressing the inulinase immobilization yield as a function of the independent variables. An empirical model based on the statistical analysis of the model parameters is presented below (Equation (1)):

I (%) = 39681.47 − 4443.74 X_1_ + 6533.19 X_2_ + 5604.3 X_1_·X_2_ (1)

where X_1_ is the enzyme concentration and X_2_ is the adsorbent:adsorbate ratio. It includes the significant terms (*p* < 0.10) concerning the inulinase immobilization and it was validated by analysis of variance (ANOVA) data, which is presented in Table 2. The calculated F-test for Equation (1) was about three times greater than the tabulated ones for significance at *p* = 0.10 and the determination of coefficient (R^2^) was 0.81. The high values for the determination of coefficient indicate good fitting of the experimental data, allowing the use of such a model to predict process performance. For the biotechnological process, this coefficient of determination (0.81) is acceptable because of the high variability in the bioprocess.

**Table 2:** ANOVA for inulinase immobilization.

	SS	*df*	MS	F	R^2^
Regression	623,581,895	3	207,860,631	9.84	81.0
Residual	147,736,259	7	21,105,179		
Total	771,318,154	10			

F_3;7;0.1_ = 3.07.

**Figure 2:**
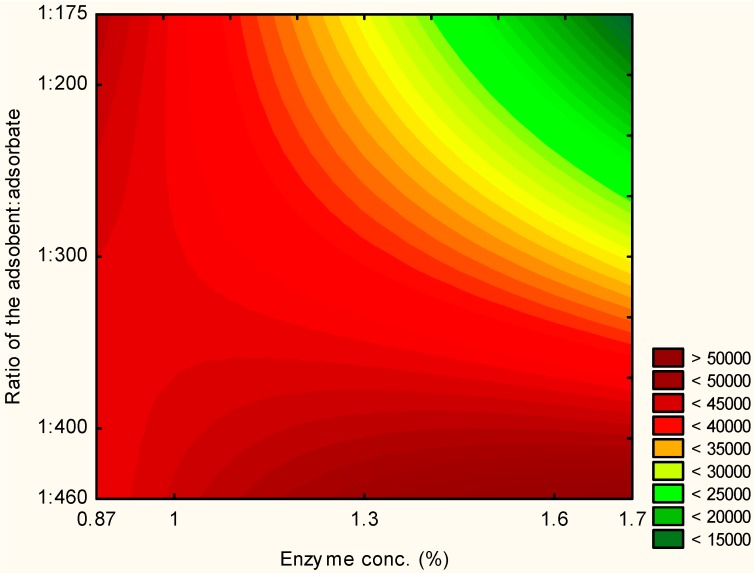
Contour diagrams for the inulinase immobilization according to the CCRD.

The validated model was used to optimize the process using the tool response/desirability profiling of the Statistica 8.0 software. The desirability of the function allows the produced response surface to be inspected by fitting the observed responses using Equation (1) based on the levels of the independent variables. This equation was used to predict the response (inulinase immobilization) value at different combinations of levels of the independent variables, to specify desirable functions for the dependent variable, and to search for the levels of the independent variables that produce the most desirable responses for the dependent variable [16]. In Figure 2 it can be observed that better responses were obtained for small enzyme concentrations and high adsorbent:adsorbate ratios (assay 3) and for a small adsorbent:adsorbate ratio and intermediate conditions for the enzyme concentrations (assay 7).

### 2.2. Thermal and Shelf Stabilities

**Figure 3:**
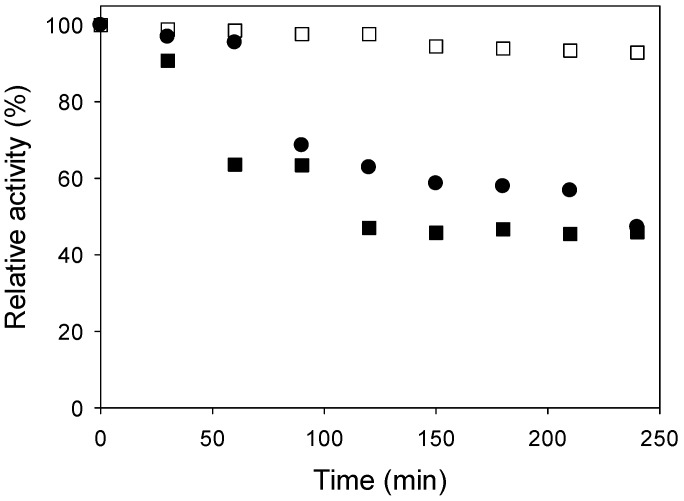
Thermal stability of immobilized enzyme ((■) 30 °C, (□) 50 °C and (●) 70 °C).

The stability studies were done with a 1.3% (v/v) enzyme concentration and a 1:400 adsorbent:adsorbate ratio. The optimum temperature of the immobilized enzyme on carbon nanotubes was 50 °C. According to Figure 3, it is clear that at a temperature of 50 °C the relative activity of the immobilized enzyme was considerably higher compared to the other temperatures studied (30 and 70 °C). Other authors found similar results concerning the best temperature for the thermal stability. Rocha* et al.* [17] achieved the maximum activity for immobilized inulinase on Amberlite IRC 50 at 50 °C. Danial* et al.* [18] and Yewale* et al.* [6], found 60 °C to be the best temperature for immobilized inulinase on grafted alginate and chitosan, respectively. Richeti* et al.* [19], observed an optimum value at 55 °C for immobilized inulinase on sodium alginate. 

The results (Figure 3) revealed that at 50 °C and after four hours, the enzyme could retain 93% of its initial activity, while at the other evaluated temperatures (30 and 70 °C) the relative activity was 45% and 47%, respectively, after 240 min. The enzyme probably demonstrated the best thermal stability at 50 °C because the enzyme is activated in this temperature; the inulinase activity is assayed in this temperature too. 

In this work the enzyme immobilization maintained its activity during five weeks, while on the other hand, this was not observed for the free enzyme. In the literature some have related that the immobilization [6] and the CNTs are responsible for the increased thermal stability [20].

**Figure 4:**
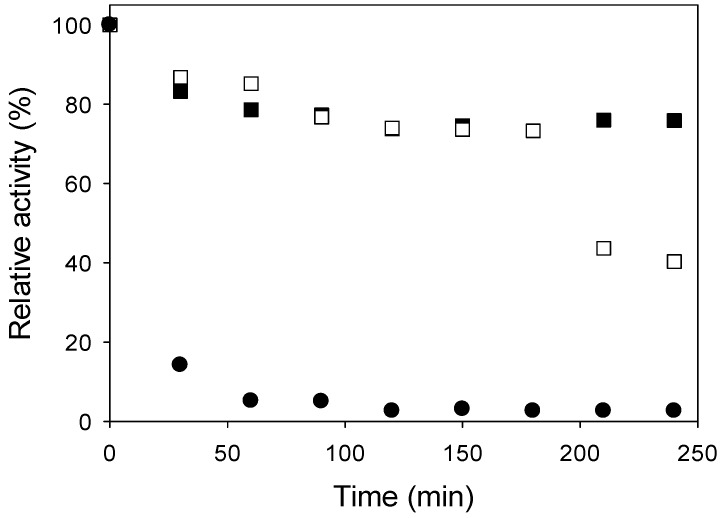
Thermal stability of free enzyme ((■) 30 °C, (□) 50 °C and (●) 70 °C).

Figure 4 presents the results of thermal stability of free inulinase at 30, 50 and 70 °C. According to the results the free inulinase lost 60% of its initial activity after 240 min at 50 °C, whereas under identical conditions the immobilized enzyme was more stable and retained 93% of its initial activity. Similar results were reported for inulinase from *Aspergillus niger* NCIM 945 immobilized on chitosan [6]. At 70 °C, the highest temperature studied, after 240 min practically all initially activity was lost; these results demonstrated that the immobilization improved the stability at the high temperatures studied. This enhanced thermal stability after the immobilization is advantageous for the process scale-up [6].

**Figure 5:**
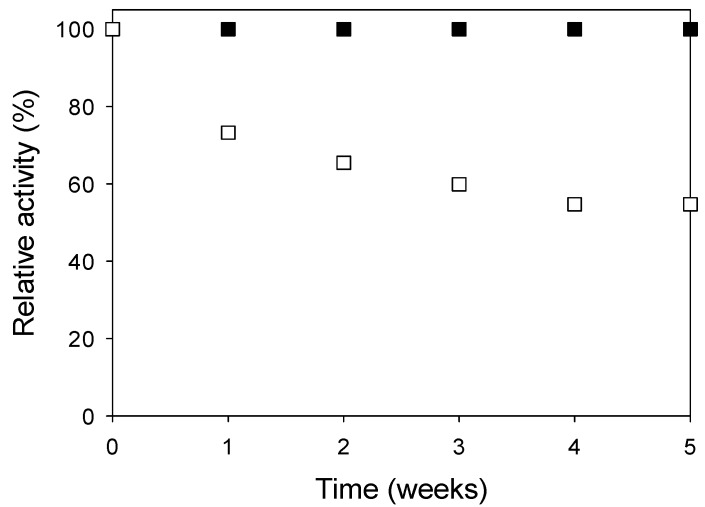
Shelf stability of immobilized (■) and (□) free inulinase during storage at room temperature.

The data shown in Figure 5 indicates that the immobilized enzyme retained 100% of its activity after five weeks of storage at room temperature. However, the storage stability can be improved at lower temperatures, e.g., 4–5 °C. The free enzyme lost practically half of its activity at room temperature after 35 days; the relative activity is that case was only 54.7%.

## 3. Experimental Section

### 3.1. Materials

Commercial inulinase which was obtained from *Aspergillus niger* (fructozyme, exo-inulinase EC 3.2.1.80 and endo-inulinase EC 3.2.1.7) was purchased from Sigma-Aldrich (São Paulo, Brazil). The multiwalled carbon nanotubes (88% purity, 50–80 nm and 10–20 μm length) were purchased from NanoAmor (Houston, TX, USA) and others reagents from Vetec (Rio de Janeiro, Brazil).

### 3.2. Enzyme Immobilization

Adsorption experiments were carried out to investigate the effect of process variables on the inulinase immobilization from aqueous solution. The variables investigated in this work were the enzyme concentration (0.8%–1.7% v/v) and the adsorbent:adsorbate ratio (1:460–1:175) at 25 °C.

The adsorption of inulinase was performed by using a batch technique. Typically, carbon nanotubes (0.025 g) were placed in Erlenmeyers flasks containing of inulinase solution in sodium acetate buffer (pH 4.8) at the desired concentration. The resulting solution was maintained under agitation (150 rpm), then an aliquot of the aqueous solution was taken at various time intervals and filtered through a polyvinylidene difluoride (PVDF) membrane (0.22 μm) before analysis. The inulinase activity in the aqueous solution was determined according to procedure defined below.

### 3.3. Inulinase Activity Assay

An aliquot of the enzyme (0.5 g) was incubated with sucrose solution (4.5 mL, 2% w/v) in sodium acetate buffer (0.1 M, pH 4.8) at 50 °C. Released reducing sugars were measured by the 3.5-dinitrosalicylic acid method [21]. A separate blank was set up for each sample to correct for the non-enzymatic release of sugars. One unit of inulinase activity was defined as the amount of enzyme necessary to hydrolyze 1 μmol of sucrose per minute under the mentioned conditions (sucrose as a substrate). The adsorption (inulinase immobilization) was determined using Equation (2).

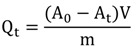
(2)
where A_0_ and A_t_ (U·mL^−1^) are the inulinase activities at the t = 0 and time t, respectively, V (mL) is the volume of solution, and m (g) is the mass of carbon nanotubes.

### 3.4. Experimental Design

The effects of enzyme concentration (0.8%–1.7% v/v) and the adsorbent:adsorbate ratio (1:460–1:175) were assessed by means of a central composite rotational design (CCRD) for two independent variables. Table 1 presents the range of investigated variables. All the results were analyzed using the software Statistica^®^ 8.0 (Statsoft Inc., Tulsa, OK, USA).

### 3.5. Thermal Stability

The stability was determined by incubation of immobilized and free enzymes in 0.1 M acetate buffer (pH 4.8) without substrate at 30, 50 and 70 °C. Samples were taken at different intervals during 4 h and the inulinase activity was determined. The relative activity at each temperature was determined by taking the activity at week 0 as 100%.

### 3.6. Shelf Stability

The shelf stability was determined by incubation of immobilized and free enzymes in 0.1 M acetate buffer (pH 4.8) without substrate at room temperature (25 °C). Samples were taken at different intervals and the inulinase activity was determined. The relative activity at each temperature was determined by taking the activity at week 0 as 100%.

## 4. Conclusions

Inulinase could be successfully immobilized using multiwalled carbon nanotubes. In the CCRD the optimal immobilization strategy parameters were 1.3% (v/v) enzyme concentration and a 1:460 adsorbent:adsorbate ratio. The optimum temperature in the thermal stability tests was 50 °C and the inulinase immobilization retained 93% of its initial activity after 240 min. The immobilized inulinase maintained 100% of its activity during five weeks at room temperature. In the best conditions identified by the CCRD the enzyme loading capacity was 51,047 U/g, which indicates that the inulinase immobilization on carbon nanotubes is a promising technique.
